# Investigation of acute organophosphate poisoning in humans based on sociodemographic and role of neurotransmitters with survival study in South India

**DOI:** 10.1038/s41598-022-21054-1

**Published:** 2022-10-03

**Authors:** Sukesh Narayan Sinha, Rajesh Kumar Kumpati, Pandu Naik Ramavath, Rajendra Sangaraju, Balaji Gouda, Priyanka Chougule

**Affiliations:** 1 Division of Food Safety, Indian Council of Medical Research - National Institute of Nutrition, Tarnaka, Hyderabad, 500007 India; 2 Department of Anaesthesia, Osmania General Hospital and Osmania Medical College, Hyderabad, 500012 India

**Keywords:** Environmental sciences, Health occupations, Risk factors

## Abstract

The aim of this study was to investigate the sociodemographic characteristics of patients based on the poison chosen and different types of organophosphorus compounds. The data were collected to explore the sociodemographic characteristics of organophosphate (OP)-poisoned patients based on the source, site, and route of poisoning, education level, occupational status, and the purpose of poisoning. Furthermore, we estimated the serotonin and dopamine levels in the plasma samples of patients, and survival plots were also described. During the study of OP pesticide poisoning in 116 human subjects and 5 healthy volunteers, we observed, based on the survival plot, that75.9% of the patients were discharged, and the remaining patients died (24.1% of the patients) due to respiratory failure followed by cardiac arrest. Our findings suggest that the serotonin levels significantly (*p* < 0.01 and *p* < 0.001) decreased from 12 to 36 h, whereas the dopamine levels slightly increased from 12 to 36 h in the group with OP poisoning compared to the control group. Based on these findings, this study may aid in deciphering the precise mechanism by which pesticides cause behavioural changes that influence serotonin and dopamine levels in OP-poisoned patients. The purpose of this work was to serve as a small reminder of the risk to public health associated with organophosphate pesticides.

## Introduction

Suicidal and intentional self-harm are severe public health issues, especially in middle—and low-income nations^1^. Suicide reports from poorer countries imply that the acts of suicide and self-harm are ill-considered, with the selection of agents made impulsively. The chosen poison is determined by its availability instead of lethality^2^. Furthermore, significant psychopathology is frequently missing in those who commit suicide^3^. While some poisonings, such as organophosphate poisoning in farmers, have been widely documented, the vast majority of reported pesticide poisonings and the socioenvironmental aspects of their use are unknown^4^. Organophosphorus (OP) compounds are a diverse collection of neurotoxic chemicals that have been widely used as insecticides and chemical weapons. Furthermore, many OP poisoning cases that led to hospital admission were the result of occupational exposure, particularly in agricultural countries^5^. The problems were more prevalent in rural areas of India. Chintale et al., 2016 found that organophosphate (OP) poisoning is widespread in southern and central areas of India, which results in a higher number of fatalities^6^. Because pesticides are commonly available and easy to obtain, they are frequently used for self-harm, i.e., causes of suicide in humans due to the liberalization of regulatory agencies in developing countries compared to developed countries, which maintain strict monitoring by regulatory agencies^7^. The percentage of people in India who commit suicide by poisoning themselves with OPs varies greatly, ranging from 10.2 to 43.87%^8^. Currently, most regulatory agencies, such as the Federal Insecticide Fungicide and Rodenticide Act (FIFRA; http://www.epa.gov/pesticides/bluebook/FIFRA.pdf), the Federal Food Drug and Cosmetic Act (FFDCA;http://www.epa.gov/pesticides/regulating/laws.htm#ffdca)and the U.S. Environmental Protection Agency (EPA; http://www.epa.gov/pesticides/fees/tool/category-table.html) regulate the usage of pesticides to protect public health. In the past 30 years, a number of investigations have reported that pesticides, particularly the organophosphate group, are responsible for the admission of millions of people suffering from accidental poisoning, as well as suicidal patients, to hospitals^9^. There are few or no programs to control OP exposure in so many developing nations. As a result, it has been estimated that up to 25 million agricultural workers globally unintentionally poison themselves with pesticides each year^10^. Pesticide exposure has been linked to neurological illnesses, such as multiple sclerosis, Alzheimer's disease, and Parkinson's disease, in several epidemiological studies^11,12^.

Costa, 2006,reported that the effect of OPs on the inhibition of acetylcholine esterase (AChE) leads to the elevation of acetylcholine (Ach) at autonomic nerve synapses^13^. Other neurotoxic pathways, such as the dopaminergic and serotoninergic pathways, which are not dependent on AChE inhibition, may also be used to harm the brain. Behavioural and functional abnormalities have been connected to changes in the dopaminergic system, which have been linked to delayed neurotoxicity^14^. Pesticide exposure has been revealed as a potential cause of severe depression, which, if handled early enough, could minimize the incidence of suicide among agricultural workers^15^. Researchers have examined probable causal links between exposure to pesticides and stress, impulsive suicidal thoughts, and suicide^16,17^. Saravi and Dehpouret., 2016 hypothesized that environmental and genetic factors play a role in the pathophysiology of neurobehavioural and neurodevelopmental disorders^18^. According to previously reported studies, specific depressive symptoms were more common in people who had been poisoned with pesticides, and these symptoms were associated with an increased risk for agriculture workers^19,20^. Apart from AchE's role in OP poisoning cases, we hypothesized, due to the alteration of dopamine and serotonin levels in acute OP poisoning cases, that unintentional and intentional pesticide exposure also causes brain damage through cognitive impairments and behavioral and neurodegenerative diseases in humans. The objectives of this study were to investigate the following: the sociodemographic characteristics of OP-poisoned patients based on the poison chosen and different types of organophosphate compounds, such as chlorpyrifos, phorate, monocrotophos, and unknown OPs; the poisoning site and route; occupation; locality; education level; and the reason for poisoning. This study also trying to clarify that the effect of neurotransmitters (serotonin and dopamine) levels on the OP’s poisoning subjects as well as survival plots in the OP poisoning in human subjects.

## Results

### Descriptive and comparative analysis for individuals (N = 116) who ingested various types of OP poisonous compounds

During the study, there were 116 patients with suicide attempts as well as occupational exposures to acute OP poisonous compounds at the emergency department of Osmania General Hospital, Hyderabad, Telangana, India. The sociodemographic details of the patients who ingested different types of OP compounds are given (see Table 1). Most of patients were males and adults, and their mean age was 34.80 ± 14.82 years. A total of 116 patients were available for the prospective study and were interviewed once daily during working hours, except on public holidays, During the study period; patients who recovered from OP poisoning were discharged or referred based on the clinical picture, and few patients’ clinical data were collected from doctors in the emergency ward based on this prospective study. In this study, 116 patients who individually ingested different types of organophosphate and pyrethroid compounds were represented as a percentage of people. The most common poisons, shown in Table 1, were as follows: amitraz (2.59%), chlorpyrifos (6.90%), chlorpyrifos + cypermethrin (0.86%), cyhalothrin (0.56%), deltamethrin (0.86%), imidacloprid (0.86%), monocrotophos (22.41%), paraquat (1.72%), phorate (20.69%), and profenofos (6.90%). During the study, we observed that 75.9% of the patients were discharged, and the remaining patients died (24.1% of the patients) due to respiratory failure followed by cardiac arrest. Most of the people were exposed to monocrotophos and unknown OP compounds, i.e., (22.41% of the patients) and phorate (20.69% of the patients), whereas fewer people were exposed to chlorpyrifos (6.90% of the patients).

**Table 1:** Study description and comparative analysis of OP-poisoned individuals (N = 116) who ingested different types of OPs, based on the WHO Recommended Classification of Pesticides by Hazard.

Toxin consumed (different types of oppoisonous compounds)	No. of persons (N in %)	Males		Females		Age (Mean ± SD)	Discharged (males & females)	Mortality (males & females)	WHO classification of pesticides by hazard category
		(N)	(%)	(N)	(%)				
Acephate	3 (2.59%)	0	0.00	3	2.59	21.66 ± 2.88	2.6	0.0	Class-II
Amitrazs	1 (0.86%)	1	0.86	0	0.00	40 ± 0.0	0.9	0.0	Class-II
Chlorpyrifos	8 (6.90%)	5	4.31	3	2.59	31.3 ± 7.32	5.2	1.7	Class-II
Chlorpyrifos + cypermethrin	1 (0.86%)	0	0.00	1	0.86	30 ± 0.0	0.9	0.0	Class-II + Class-II
Cyhalothrin	1 (0.86%)	1	0.86	0	0.00	55 ± 0.0	0.0	0.9	Class-II
Deltamethrin	1 (0.86%)	1	0.86	0	0.00	18 ± 0.0	0.9	0.0	Class-II
Deltametrin + triazophos	6 (5.17%)	4	3.45	2	1.72	39.5 ± 17.59	3.4	1.7	Class-II + Class-Ib
Dichlorvos	3 (2.59%)	1	0.86	2	1.72	29.6 ± 8.9	2.6	0.0	Class-Ib
Dimethoate	3 (2.59%)	3	2.59	0	0.00	40.6 ± 12.5	2.6	0.0	Class-II
Imidacloprid	1 (0.86%)	1	0.86	0	0.00	35 ± 0.0	0.0	0.9	Class-II
Monocrotophos	26 (22.41%)	20	17.24	6	5.17	32.15 ± 14.98	17.2	5.2	Class-Ib
Monocrotophos + profenofos	1 (0.86%)	1	0.86	0	0.00	20 ± 0.0	0.9	0.0	Class-Ib + Class-II
Paraquat	2 (1.72%)	2	1.72	0	0.00	40 ± 21.12	0.9	0.9	Class-II
Phorate	24 (20.69%)	18	15.52	6	5.17	36.4 ± 15.3	15.5	5.2	Class-Ia
Profenofos	8 (6.90%)	6	5.17	2	1.72	35.2 ± 16.4	5.2	1.7	Class-II
Quinalphos	1 (0.86%)	1	0.86	0	0.00	33 ± 0.0	0.9	0.0	Class-II
Unknown OP compounds	26 (22.41%)	19	16.38	7	6.03	36.7 ± 15.3	16.4	6.0	-NA-
Total	116 (100%)	84	72.41	32	27.59	34.80 ± 14.82	75.9	24.1	

Study description and comparative analysis of OP-poisoned individuals (N = 116) who ingested different types of OPs.

Class Ia, Extremely hazardous; Class Ib, Highly hazardous; Class II, Moderately hazardous.

### Sociodemographic and case-specific data of poisoned patients (N = 116)

In this study, we analysed the sociodemographic data of the male and female subjects. Based on sex, we observed that more males (N = 84 patients; 72.41%) than females (N = 32 patients; 27.58%) had OP poisoning; based on occupational status, we found that more working males (N = 63 patients; 87.5%) and females (N = 9 patients; 12.5%) than nonworking males (N = 21 patients; 47.7) and females (N = 23; 52.3%) had OP poisoning; the majority of the poisoned patients were found to be working individuals rather than nonworking individuals. Based on the route of exposure, we observed OP poisoning occurred via the oral route in males (N = 76; 70.4%) and females (N = 32; 29.6%) and via the inhalation or dermal route in males (N = 8; 100%) and females (N = 0; 0%); the majority of the poisoning cases were from oral route exposure rather than from inhalation or dermal route exposure. From a regional perspective, we observed that poisoning occurred in males (N = 71; 77.2%) and females (N = 21; 22.8%) living in rural areas and males (N = 13; 54.2%) and females (N = 11; 45.8%) living in urban areas; the majority of the poisoning occurred in rural areas rather than urban areas. Based on the sites of poisoning, poisoning occurred at home for males (N = 46; 60.5%) and females (N = 30; 39.5%) and outside of the home for males (N = 38; 95.0%) and females (N = 2; 5%), the majority of the poisons were obtained within the home rather than outside the home. The reasons for poisoning were as follows: relationship conflicts: males (N = 47; 63.5%) and females (N = 27; 36.5%); work-related stress: males (N = 20; 90.9%) and females (N = 02; 9.1%); illness: males (N = 02; 50%) and females (N = 02; 50%); and other issues: males (N = 15; 93.8%) and females (N = 01; 6.2%). The majority of these attempts were aggressive and appeared to be in response to interpersonal issues related to family relationship conflicts. Based on the occupation, region, site of poisoning, the reason for poisoning, and reason for suicide, males were significantly more affected by OP suicide and exposure than females, except for the route of exposure (see Table 2).

**Table 2:** Sociodemographic characteristics and details of OP-poisoned patients (N = 116) between the male and females.

Sex	Males		Females		*p* value
Based on sociodemographic analysis	No. of persons (N)	Percentage (%)	No. of persons (N)	Percentage (%)	Pearson chi-square
*Occupation*					
Working	63	87.5	9	12.5	0.001***
Not working	21	47.7	23	52.3	
*Route of exposure*					
Oral	76	70.4	32	29.6	0.070^ns^
Inhalation/dermal	8	100	0	0	
*Region*					
Rural	71	77.2	21	22.8	0.025*
Urban	13	54.2	11	45.8	
*Site of poisoning*					
Home	46	60.5	30	39.5	0.001***
Outside the home	38	95.0	2	5.0	
*Reason for poisoning*					
Relationship conflicts	47	63.5	27	36.5	0.010**
Work-related stress	20	90.9	02	9.1	
Illness	02	50	02	50	
Other issues	15	93.8	01	6.2	

*p < 0.05, **p < 0.01, and ***p < 0.001, and ns; nonsignificant, by using Pearson Chi-Square analysis.

### Effect of acute OP poisoning on neurotransmitters, such as serotonin and dopamine, in humans

**Figure 1:**
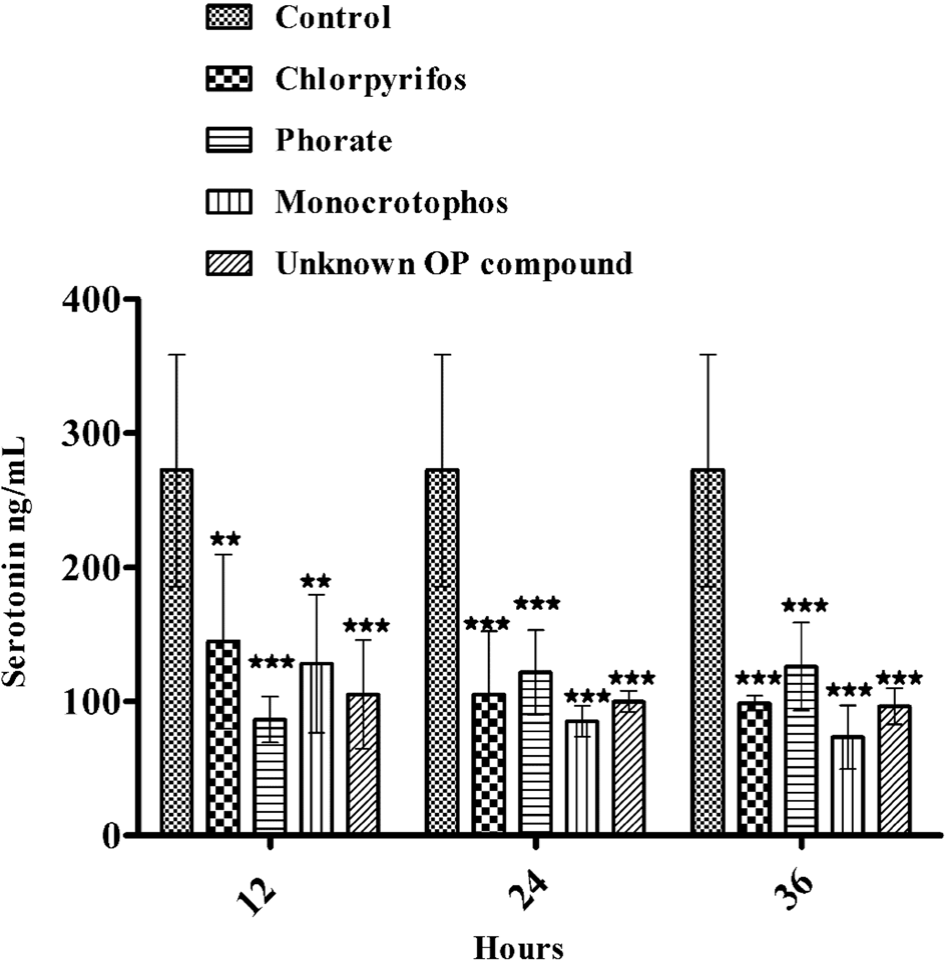
Neurotransmitter levels (serotonin) after acute OP poisoning with chlorpyrifos, phorate, monocrotophos, and unknown OP compounds; serotonin concentrations in human plasma samples from 12 to 36 h on consecutive days after admission to the hospital for acute OP poisoning; neurotransmitter levels were quantified using ELISA kits. The outcomes are presented as the mean ± SD (OP poisoning cases (N = 3) and control group non-OP exposures (N = 05)). *p < 0.05, **p < 0.01, and ***p < 0.001, compared with the control group. *p* > 0.05was considered nonsignificant.

**Figure 2:**
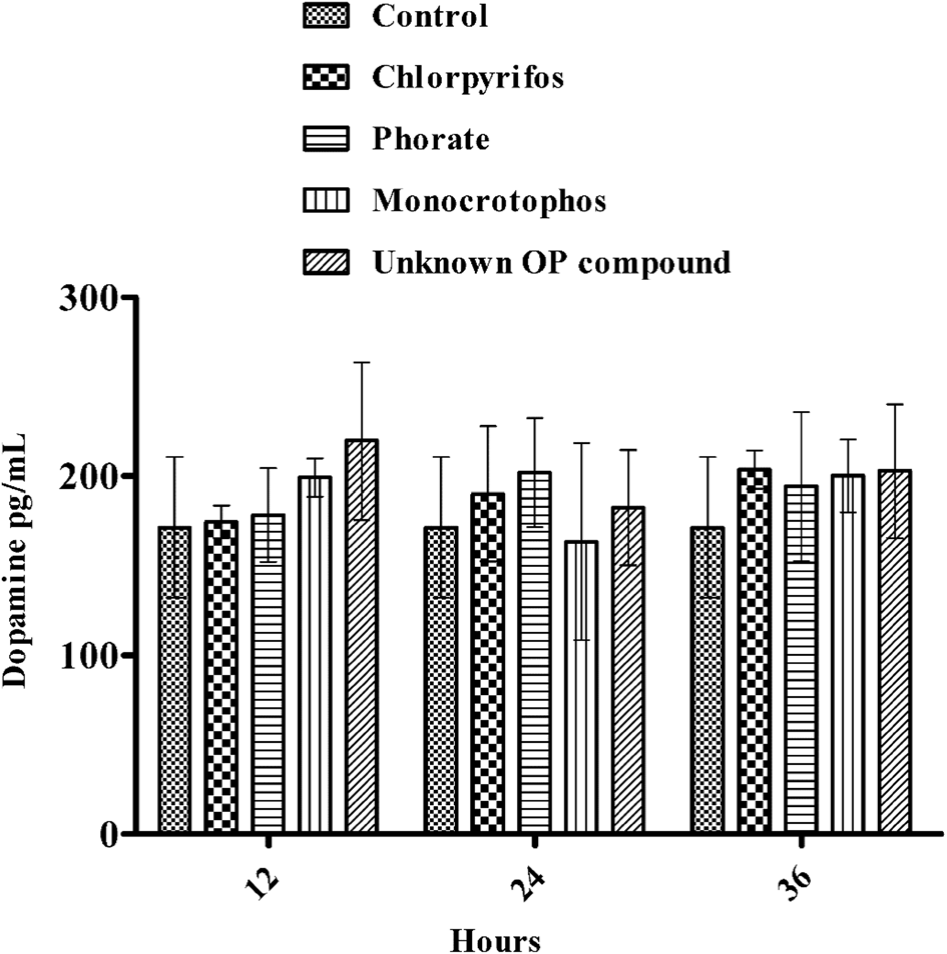
Neurotransmitter levels (dopamine levels) after acute OP poisoning with chlorpyrifos, phorate, monocrotophos, and unknown OP compounds; dopamine concentrations in human plasma samples from 12 to 36 h on consecutive days after admission to the hospital for acute OP poisoning; neurotransmitter levels were quantified using ELISA kits. The outcomes are presented as the mean ± SD (OP poisoning cases (N = 3) and control group non-OP exposures(N = 5)). *p* > 0.05was considered nonsignificant.

The effects of OP poisoning on the levels of neurotransmitters such as serotonin and dopamine in human plasma (N = 3 for each type of OP poison) were investigated in individuals exposed to different types of OP compounds, such as chlorpyrifos, phorate, monocrotophos, and unknown OP compounds, compared to healthy volunteers in the control group (N = 05), who were not exposed to OP poisoning. Especially in critically ill patients, it is challenging to alter the levels of some neurotransmitters, such as Ach, 5-HT (serotonin), and dopamine. Apart from acetylcholine, serotonin and dopamine levels play a major role in the neurobehavioural aspects of suicidal patients. Therefore, we examined these neurotransmitter levels in the plasma samples of patients poisoned with different types of OPs and compared them to those of the control group (healthy volunteers) by using ELISA techniques. Based on the results, the serotonin level was significantly (**p < 0.01, ***p < 0.001) decreased in OP-poisoned patients exposed to chlorpyrifos, phorate, monocrotophos, and unknown OP compound when compared to those of the control group from 12 to 36 h (12 h time intervals) (Fig. 1). Additionally, we measured the amount of dopamine in the plasma of people who were exposed to OPs and compared it to those of the control groups from 12 to 36 h (12-h time intervals) (Fig. 2). Based on the results, the dopamine level was nonsignificantly increased in OP-poisoned subjects exposed to chlorpyrifos, phorate, monocrotophos, and unknown OP compounds when compared to those of the control group. However, the dopamine level was slightly increased in OP-poisoned subjects exposed to chlorpyrifos, phorate, monocrotophos, and unknown OP compounds from 12 to 36 h when compared to those of the control group because of cross-linking between acetylcholine and AchE inhibition in OP-poisoned patients. There were no significant changes in serotonin and dopamine levels in the control group of individuals who were not exposed to OP poisoning between 12 and 36 h (at every 12-h time interval). These findings suggest that subtle changes in these neurotransmitter patterns might restore some vital organ functions, resulting in normal neurotransmitter homeostasis in the brain and preventing suicidal episodes. According to the findings, the majority of mortality has been seen in only four different types of OP pesticides poisoned in human subjects (Table 1) such as chlorpyrifos, phoarate, monocrotophos, and unknown OP compounds. Therefore, we selected a few patients for estimating the neurotransmitter levels in plasma exposed to these four types of OP, such as chlorpyrifos, phorate, monocrotophos, and unknown pesticides poisoning human subject groups.

### Risk factors for mortality in acute OP-poisoned human subjects

**Figure 3:**
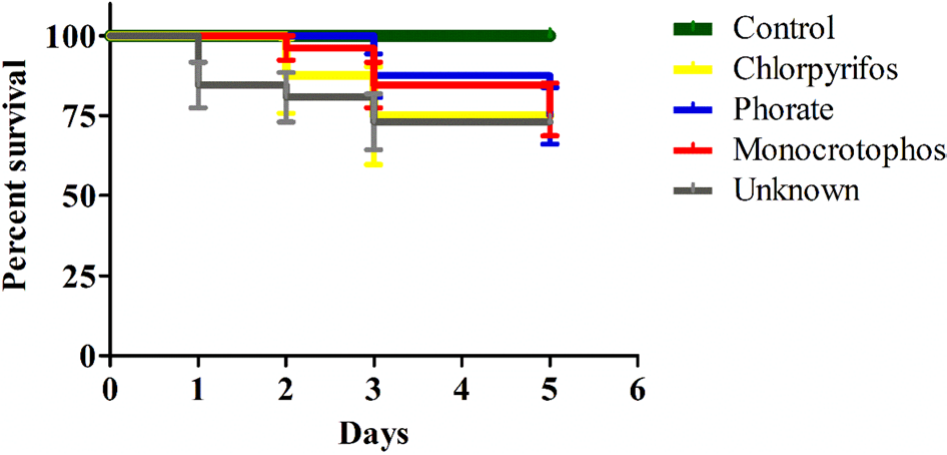
Effect of acute OP poisoning with chlorpyrifos, phorate, monocrotophos, and unknown OP compounds on a human survival plot from Day 1 to Day 5 using the Kaplan‒Meier statistics in Prism Software Version5.0.

Based on the Kaplan‒Meier survival plot for the 5-day mortality study, we observed a severe risk factor for mortality in patients with acute OP poisoning: human subjects exposed to monocrotophos and phorate had a mortality rate of 5.2% mortality; subjects exposed tochlorpyrifos, profenophos, and the combination of deltamethrin and triazophos had a mortality rate of 1.7% ; and subjects exposed to unknown OP compounds showed a mortality rate of 6.0% from Day 1 to Day 5 of admission at the general tertiary hospital. In this study, a total of 116 patients were identified as having acute OP pesticide poisoning, and the mortality rate was 24.1%, while the remaining patients survived, with a survival rate of 75.9%. In this context, the results of subjects exposed to chlorpyrifos, phorate, monocrotophos, and unknown OP compounds were compared to those of the control group (healthy volunteers). The mortality rate from Day 2 to Day 5 was studied, and we observed a mortality rate of approximately 25% (Fig. 3). Evidence suggests that subtle changes in these neurotransmitter patterns might alter some vital organ functions, leading to respiratory failure followed by cardiac arrest and resulting in death.

### Clinical aspects of OP poisoning patients

Based on the clinical diagnosis, Subjects after being admitted to the hospital, doctors first check the appearance (smell), and clinical history of the subject followed by the symptoms (SLUDGE). Before to the treatment, OP subjects will have bradycardia, and pupils are in constricted stage. If the patient is with respiratory failure, then they were treated with mechanical ventilator support till recovery.

### OP intoxication of poisoning cases

In OP poisoning cases after being admitted to the hospital, the clinician will do decontamination and gastric lavage (within 1 to 2 h) followed by treatment with atropine (0.6 mg) depending on pupil reactions and Pralidoxime (PAM) only in the early stages. Pyridostigmine will also help to reduce the secretions (SLUDGE). To prevent gastric ulceration, antacids will be given to the OP Poisoning subjects. If any symptomatic treatment is present, i.e., respiratory failure, patients were treated with intubation followed by mechanical ventilation support.

## Discussion

According to the WHO in 2012, approximately 3 million OP poisoning cases occur each year due to both unintentional and intentional exposures, resulting in approximately 300,000 deaths per year^21^. According to previous reports, overstimulating cholinergic receptors (AChRs) can disrupt the balance of glutamatergic, GABAergic, and dopaminergic neurotransmission, resulting in neurotoxicity which effects on the cholinergic system and altered neuronal functions in the brain. The imbalance between acetylcholine and dopamine in the striatum nigra leads to Parkinson’s disease and influences CNS-related disorders such as Alzheimer's disease and multiple sclerosis^11,12^. The toxic effects of OP pesticides typically induce the suppression of acetylcholinesterase (AChE) enzymes, which results in elevated acetylcholine levels in autonomic nerve synapses, leading to salivation, in addition to other symptoms, and the overstimulation of acetylcholine receptors in the nervous system(NS)^13^. Organophosphate (OP) chemicals can cause major pesticide-related illnesses and deaths in underdeveloped nations, such as India. Studies have proven that pesticide exposure has significant detrimental effects on human health. Acute OP poisoning results in death or a variety of chronic health disorders in humans^22,23^. Our study team previously reported the toxicokinetic analyses of widely used OP pesticides of the same acute OP-poisoned patients. In the aforementioned study, our research group focused on pesticide concentration as well as AchE inhibition activity in the human blood samples^24^. Organophosphate poisoning can have a considerable negative impact on morbidity and mortality. Organophosphates, often known as nerve agents, cause seizures that develop in three stages. As a result of overstimulation of the cholinergic system, the first five minutes of OP exposure cause seizures. If the patient’s intoxication with OP poisoning which inhibits the AchE enzyme activity leads to the availability of acetylcholine at the nerve synapse leads to seizure activity. Agents with central anticholinergic effects can stop or delay these seizures during this time. Other alterations, such as lower brain norepinephrine levels, an enhanced glutaminergic response, and NMDA receptor activation, are seen after 5 min of OP exposure. Anticholinergic therapy alone will not stop seizures in this mixed cholinergic and noncholinergic stage. Noncholinergic pathways are involved in the continued seizure activity 40 min after OP exposure, which causes structural neuronal impairment that is challenging to treat with medication. It's crucial to keep in mind how nicotinic overstimulation affects the neuromuscular junction when treating people who have been poisoned by organophosphates. Muscle fasciculations, weakness, and outright paralysis may be seen in patients. Seizures might not be obvious in this environment. As a result, until it can be shown otherwise, people who have been exposed to organophosphates and seem to be unresponsive and flaccid should be thought to be having seizures. In these situations, aggressive seizure control (with atropine and benzodiazepines), EEG (electroencephalogram) monitoring, and pralidoxime must be started right away. If any symptomatic treatment is there the respiratory failure, patients were treated with mechanical ventilatory support for oxygen supply till recovery via intubation procedures. Patients were unconscious after consuming OP poison, so we did not get information regarding how much they consumed the OP compounds, but we did not get information regarding the name and some of the OP poison and we determined the concentration of OP pesticides present in poisoning blood samples collected from poisoning patient from Osmania Hospital, Hyderabad, India by using the LC–MS/MS technique, it was earlier reported in my research article^24^. Chronic and acute organophosphorus exposure has been linked to a variety of disorders in cognitive, psychomotor, and emotional behaviours, including depression, attentiveness, cognition, anxiety, and irritability deficits^25^. Additionally, studies have linked both dopamine and serotonin as being involved in depressive states and a person's ability to deal with suicidal behaviours on the way to committing suicide^26^. The significance of the serotonergic system in depression has been revitalized, and different correlations of this system with impulsive aggression, obsessive–compulsive disorder, anxiety, eating disorders, and suicidal behaviour have been identified^27^. Although serotonin and dopamine cannot be transported across the blood‒brain barrier, they are transported across the abluminal membrane surface of the blood‒brain barrier into endothelial cells, where they affect regulatory function; however, they do not cross the blood‒brain barrier since they do not cross the luminal membrane^28^. Abnormal whole blood serotonin levels have been correlated with clinical depression, and elevated plasma serotonin levels have been indicated as markers for psychopathology and suicidality^28,29^. Pesticide poisoning was responsible for 30% of the global suicide fatalities in 2007, in addition to the large majority of suicide emergency care admissions in developing countries^20,30,31^. Despite having higher pesticide sales, developed countries had pesticide fatality rates that were many times lower than those of developing countries, implying unsafe use and higher toxicity of the agents used in developing countries^32^. In the sociodemographic analysis, the results suggest that males are more likely than females to be poisoned by pesticides. However, males get pesticide poisoning from outside the home because male labourers work in the fields throughout the day in agricultural communities, and females get pesticide poisoning from within the home because women take care of the household while watching mass media, with factors such as films and television series that dramatize morbid suicide thoughts^33^. This trend might indicate that the poison chosen reflects its proximity and rapid availability during times of vulnerability. Based on locality and the availability of OP compounds, suicide attempts are more likely to occur in rural areas than in urban areas. Targeting suicide would thus necessitate both broad macroeconomic interventions, such as further study, meeting fundamental human needs, and providing population-based psychological therapies for family members, in addition to the development of intervention programs aimed at susceptible communities, such as limiting the availability of pesticides in rural areas with a minimum pesticide list and limiting media impacts of eyewitness depictions of suicide^34^. Low education has been linked to a range of direct and indirect suicide stressors, and it is a dividing line between industrialized and developing countries. Economic disadvantage, unemployment, a lack of avenues for expressing and addressing problems, and a lack of health care access are examples of indirect stresses. Illiterate individuals were almost more likely than literate individuals. Pesticide use is more of a targeted special message or circumstance manipulation for one’s benefit than a true wish to terminate life in reaction to a stressful experience, relationship strife, domestic violence, and drunkenness, which are all typical triggers^35^. There are more suicide attempts per year due to occupational proximity and relationship conflicts between family members, than any other financial crisis (such as business loss).

Pesticides such as monocrotophos, phorate, profenophos, chlorpyrifos, a combination of deltamethrin and triazophos, and unknown OP compounds showed more severe mortality than other pesticides in human subjects in this study. During the study, 116 patients with suicide attempts and unintentional exposures to acute OP poisoning compounds were observed and followed up at the emergency department of Osmania General Hospital, Hyderabad, Telangana. We analysed the sociodemographic characteristics based on sex; we observed that males (N = 84 patients; 72.41%) had more significant effects from OP poisoning than females (N = 32 patients; 27.58%). As a result, the serotonin levels significantly decreased and the dopamine levels non-significantly increased up to 36 h in OP-poisoned patients compared to the control group of healthy volunteers. During the study, we observed that 75.9% of the patients were discharged, and in the remaining patients, 24.1% died due to respiratory failure followed by cardiac arrest. Moreover, in the current study, mortality was observed from Day 2 to Day 5, with major mortality seen on the 3rd day after exposure to OPs, and we observed a mortality rate of approximately 25% in the whole study due to the availability of major OP compounds such as monocrotophos, phorate, profenophos, chlorpyrifos, and triazophos in developing countries. Evidence suggests that subtle changes in this lack of understanding of the hazards of poisonous pesticides, as well as their proper usage and storage, would pose a greater immediate risk and might alter some vital organ functions, leading to respiratory failure followed by cardiac arrest, resulting in death. The findings indicated that knowing which type and amount of pesticide were consumed on early admission, being cared for by health care professionals, followed by early diagnosis and treatment, improves patient survivability.

In this study, our results suggest significantly decreased plasma serotonin levels and slightly increased dopamine levels in OP-poisoned patients compared to healthy volunteers, suggesting subtle changes in these neurotransmitter patterns in the nervous system that lead to neurobehavioural changes in humans. Humans exposed to pyrimethamine and other insecticides have been linked to lower serotonin levels in the nervous system, which could be associated with decreased serotonin synthesis and the destruction of serotonergic neurons^36^. Because serotonin and dopamine cannot be transported across the blood‒brain barrier, it is difficult to assess behavioural and functional changes in OP-poisoned patients^37^. Although according to previous reports, abnormal levels of neurotransmitters or neurotransmitter metabolites in the cerebrospinal fluid (CSF) or plasma of patients have been interpreted as indicating abnormal brain functions, with examples including high or low plasma serotonin levels as markers for psychopathology and suicidality^28,38,39^. Hence, in this study, observations of these low plasma serotonin levels might be indicated as markers for psychopathology and suicidality. Based on the OP pesticide treatment there will be no interference with the neurotransmitters. Our results suggested that there is no increase of the levels of dopamine after giving the OP poisoning treatment. But however reported result suggested that after treatment with dopamine (1.5 μg/kg/min) and atropine (4 mg/h) to return the heart rate normotensive of OP-poisoned patients^40^. Hence, health care professionals may focus their drug regimen choice on the imbalance between serotonin and dopamine levels during the treatment period of OP-poisoned patients, which might help to restore the levels of serotonin and dopamine and maintain the homeostasis for neurotransmitters in the nervous system. A minimal pesticide list^41^ and the prohibition of illegal poison distribution activities against pesticide regulatory agencies^42^. Many countries now have credible evidence that national bans on dangerous pesticides have reduced suicide deaths while having no negative impact on agricultural productivity^43^. However, in nations with the largest incidence of suicide fatalities due to pesticide poisoning, urgent efforts are required to enhance the recognition and documentation of pesticides implicated in poisoning incidents based on hospital records and the monitoring of suicidal patients with OP poisoning. Furthermore, adequate quality surveillance is required to monitor and support public health reactions to the prospective replacement of OP chemicals through pesticide regulations. This study mainly focused on understanding the details of neurotransmitter abnormalities, such as serotonin and dopamine, in people who have been exposed to pesticides for a long time, leading to neurobehavioural changes. The research could lead to new ideas regarding neurobehavioural toxicity and new possibilities for regulatory authorities to develop relevant road maps for pesticide use, which could include risk evaluations to restore the neurotransmitter levels in OP-poisoned patients, which help to maintain mood and other behavioural alterations. Therefore, clinicians should focus on treatment and maintain the imbalance of these neurotransmitters, including serotonin and dopamine. Farmers were previously advised to keep their insecticides safely, but this policy has not been shown to be successful. Pesticide registrars and regulators play a large part in preventing suicide because limiting access to dangerous pesticides protects the public's health.

## Conclusion

In humans, death due to suicide poisoning is caused by a combination of agent, host, and environmental conditions, such as poison availability and education. Specific poisoning patterns are more common in certain demographic niche groups and are dependent on them. Suicide prevention requires both broad-based and customized approaches. Based on our findings, organophosphate poisoning continues to be a serious public health concern, particularly in underdeveloped nations, where it is the leading cause of morbidity and mortality. The results of poisoning were strongly correlated with sociodemographic factors; males were more significantly affected by OP poisoning than females based on sex, occupational status, etc. Rural communities were more affected by OP poisoning than urban communities. Strategies to lower the frequency of intentional self-poisoning fatalities in India must take restrictions on the availability of highly lethal poisons in rural communities into consideration. This study found that the serotonin levels in OP-poisoned humans were significantly lower than those in healthy volunteers. The measurement of OP, serotonin, and dopamine levels in the blood are not typically available in normal clinical practice, and ventilatory assistance is available only in large institutions in these nations, making the facilities for the management of these patients less than ideal. Doctors and general practitioners should be aware of this issue and be capable of identifying it early because it can be fatal if not properly identified and treated. Hence, medical professionals should focus on the neurotransmitter (serotonin and dopamine) homeostasis balance in OP-poisoned patients. Local health authorities urgently need to take regulatory and educational measures to stop the rising incidence of poisoning with these substances. Educating farmers on safe OP compound storage, usage, and disposal, along with tougher regulations for their sale and distribution, may lower the occurrence of OP poisoning, and as a result, the mortality rate should decrease. Public awareness of the symptoms of toxicity must be promoted. The most frequently used poisoning-related chemicals were organophosphate pesticides and insecticides. The government should create regulations to control the usage, distribution, and transportation of pesticides and insecticides. The purpose of this work was to serve as a small reminder of the risk associated with organophosphate pesticides on public health.

## Materials and methods

### Design of the study site

This research study carried out with OP-poisoned patients at Osmania General Hospital in Hyderabad, Telangana, India, which serves a mixed rural and urban population (approximately 1,000,000 people within a 100-km radius). The hospital operates a 24-h emergency room that treats a wide range of critical illnesses.

### Selection of pesticides

Organophosphate pesticides, pyrethroids, and neonicotinoid compounds, such as acephate, amitraz, chlorpyrifos, cypermethrin, cyhalothrin, deltamethrin, dichlorvos, dimethoate, imidacloprid, monocrotophos, paraquat, phorate, profenofos, triazophos, quinalphos, and unknown OP compounds, which are commonly used in and around Hyderabad, Telangana, India, were studied.

### Selection of subjects

The National Institute of Nutrition Ethics Committee (ECR-351/Inst/AP/2013) authorized experimental protocols to collect blood samples (9/I/2016) from healthy and poison-exposed subjects to conduct the present study. All the methods were performed as per the standard ethical guidelines and regulations made by the Indian Council of Medical Research-Institutional Ethics Committee for Human Subjects (https://ethics.ncdirindia.org/asset/pdf/ICMR_National_Ethical_Guidelines.pdf). Informed consent was obtained from all the subjects/guardians before the collection of the blood samples.

### Method of patient selection and survey

In this study, the inclusion criteria is the participants who has intentionally took the OP poisoning to make them suicide and admitted in Osmania General Hospital. The exclusion criteria is the subject who did not take OP pesticide. Total 121 study participants were divided into two groups, such as control individuals (healthy volunteers) (N = 05, mean age 37 ± 9.7 years) who were not exposed to OP pesticides and patients (N = 116, mean age 34.80 ± 14.82) who were exposed to organophosphate pesticides and fulfilled the following inclusion criteria were included in a once-daily cross-sectional screening of hospitalized patients in the emergency room: (1) patients poisoned by organophosphate compounds; (2) patients hospitalized within four days of ingestion and (3) preferred both sexes patients i.e., male and female. It was decided to obtain informed consent. Patients and their family members were interrogated in-depth using a survey method proforma to gather the following information: (a) sociodemographic factors; (b) occupational, social, and environmental characteristics; (c) the causes and circumstances of the OP poisoning; (d) the name and nature of the ingested OP poison; and (e) in the doctor's pre-admission hospital counselling, the patient or patient attendants were unable to identify the precise name of the pesticides due to most of the patients were illiterates: and based on the patient's clinical history of the OP poisoned subjects followed by the symptoms such as *SLUDGE* (salivation, lacrimation, urination, diaphoresis, gastrointestinal upset, emesis) etc., and as well as in OP poisoning patients Ach levels were increased in the blood due to inhibition of AchE activity which leads to increased salivation in the OP poisoned patients and also we estimated the presence of OP pesticides and AchE activity were found in blood samples OP poisoned subjects by using the LC–MS/MS techniques^24^. But some OP pesticides where not identified by LC–MS/MS therefore, we considered them to be labelled as “*unknown OP compounds*”*.* The prospective observational study collected the data of hospitalized patients for acute exposure to OP and pyrethroid pesticides, with a detailed identification of the exact chemical used, based on interviews with patients, doctors, or patient attendants. In this study, we focused mainly on sociodemographic data analysis as well as the role of neurotransmitters such as serotonin and dopamine in human plasma as well as human subject surveillance and deaths by suicide attempts and unintentional exposure to different types of OP compounds in agriculture fields.

### Sample collection

On the day of admission, blood samples were collected from the median-cubital vein into a tube containing a sodium EDTA solution made with 2 mL BD Vacutainer K2 EDTA 3.6 mg (REF 367841, USA). Blood samples from acute OP-poisoned patients were obtained at 12-h intervals for up to 60 h. The plasma samples were separated by centrifugation at 1000xg for 15 min at 2–8 °C. The supernatant from each sample was collected and stored at − 80 °C to carry out further analysis, such as ELISA techniques for the estimation of serotonin and dopamine levels.

### Estimation of neurotransmitters (dopamine and serotonin) in plasma

The plasma concentrations of neurotransmitters (dopamine and serotonin) were measured with commercially available ELISA kits. We procured a universal serotonin ELISA kit (Catalogue No: E‒E-0033 Lot No: JURBQR2YV4) and a universal dopamine ELISA kit (Catalogue No: E‒E-0046 Lot No: 4MLCYU4JPG) from Elabscience, USA. These neurotransmitters were analysed according to the instructions of the manufacturers. The assay principle is a competitive ELISA with colorimetric detection, performed using an ELISA multimode microplate reader (Synergy H1 hybrid reader, Biotek) at 450 nm.

### To demonstrate the survival of acute OP-poisoned patients

Based on the mortality rate, the ward and doctors in charge were observed and recorded with the inquiry of mortality in a general tertiary hospital in Hyderabad. The data were collected from patient admission and discharge records of acute OP-poisoned patients from Day 1 to Day 5, and the survival percentage of these studies with mortality over a 5-day period was calculated statistically using Kaplan‒Meier plots.

## Statistical analysis

All statistical analyses were carried out using SPSS statistical software version 23.0, GraphPad Prism 5.0 software, and one-way ANOVA. Compared to the normal control group (healthy volunteers), comparisons between groups with poisoning by different OPs were made using post hoc Dunnett's multiple comparison procedures for neurotransmitter estimation, and the Pearson chi-square test was used for sociodemographic data distribution between males and females. The Kaplan‒Meier comparison procedure was used for survival plots in this study. The mean and standard deviation are used to express the results. Statistical significance was considered when the *p* value was less than 0.05.

## Data Availability

All data related to this publication are available in the article.
